# Recent Advances of Magnetic Nanomaterials in Bone Tissue Repair

**DOI:** 10.3389/fchem.2020.00745

**Published:** 2020-09-25

**Authors:** Daoyang Fan, Qi Wang, Tengjiao Zhu, Hufei Wang, Bingchuan Liu, Yifan Wang, Zhongjun Liu, Xunyong Liu, Dongwei Fan, Xing Wang

**Affiliations:** ^1^Department of Orthopedic, First Affiliated Hospital of Zhengzhou University, Zhengzhou, China; ^2^Department of Pediatrics, Peking University Third Hospital, Beijing, China; ^3^Department of Orthopedic, Peking University Third Hospital, Beijing, China; ^4^Beijing National Laboratory for Molecular Sciences, State Key Laboratory of Polymer Physics & Chemistry, Institute of Chemistry, Chinese Academy of Sciences, Beijing, China; ^5^University of Chinese Academy of Sciences, Beijing, China; ^6^CED Education, North Carolina State University, Raleigh, NC, United States; ^7^School of Chemistry and Materials Science, Ludong University, Yantai, China

**Keywords:** magnetic nanomaterials, bone tissue repair, magnetic field, magnetic particles, stem cells, tissue engineering scaffolds

## Abstract

The magnetic field has been proven to enhance bone tissue repair by affecting cell metabolic behavior. Magnetic nanoparticles are used as biomaterials due to their unique magnetic properties and good biocompatibility. Through endocytosis, entering the cell makes it easier to affect the physiological function of the cell. Once the magnetic particles are exposed to an external magnetic field, they will be rapidly magnetized. The magnetic particles and the magnetic field work together to enhance the effectiveness of their bone tissue repair treatment. This article reviews the common synthesis methods, the mechanism, and application of magnetic nanomaterials in the field of bone tissue repair.

## Introduction

Bone is a natural complex of inorganic and organic materials (Abou Neel et al., 2016; Loi et al., 2016; Wang et al., 2020). The main component of the inorganic material is crystalline hydroxyapatite while the organic substance is mainly fibrous collagen (Loi et al., 2016). Bone has the ability to regenerate and repair itself. Although for bone defects caused by external damage or due to bone diseases, tumors, and abnormal bone growth, the self-repairing ability of the bone alone cannot achieve the purpose of healing (Loi et al., 2016; Michalski and McCauley, 2017). It is necessary to resort to medical materials including autologous bone tissue, allogeneic bone tissue, and bone tissue substitutes. Either method requires the regeneration of local bone tissue. In this process, external stimulations including stress stimulation, chemical stimulation, biological factor stimulation, magnetic field, and electric field are considered as necessary conditions (Abou Neel et al., 2016; Loi et al., 2016; Michalski and McCauley, 2017; Debnath et al., 2018).

The magnetic field has been proven to enhance bone tissue repair by affecting cell metabolic behavior (Iwasa and Reddi, 2018; He et al., 2019; Liu H. Y. et al., 2020). In recent research, iron is the most common material used with its para-magnetism (Luo et al., 2018; Xia et al., 2018; Yu et al., 2019). The unpaired electrons of the outermost layer spin to make the atom maintain a certain magnetic moment. This atomic magnetic moment is arranged along the magnetic field under the action of an external magnetic field, showing a weak magnetic force that conforms to the magnetic field (Iwasa and Reddi, 2018; Luo et al., 2018). This substance is called paramagnetic substance. Ferromagnetic substances have atomic magnetic moments composed of unpaired spin electrons (Iwasa and Reddi, 2018). In the absence of a magnetic field, the atomic magnetic moments are also neatly arranged, showing strong magnetism to the outside (Luo et al., 2018; Xia et al., 2018; He et al., 2019). Containing a small amount of paramagnetic iron, the bone is magnetically conductive (Luo et al., 2018; Xia et al., 2018; Yu et al., 2019).

Magnetic nanoparticles (MNPs) are used as biomaterials due to their unique magnetic properties and good biocompatibility (Aliramaji et al., 2017; Brett et al., 2017). Recently, they have been widely applied in drug transportation, magnetic hyperthermia, nuclear magnetic imaging, and biological separation (Li et al., 2016; Jia et al., 2019; Nejadnik et al., 2019). The magnetic particles are slowly deposited on the surface of the cell membrane under the action of the magnetic field. The cells engulf the magnetic particles through endocytosis. Entering the cell makes it easier to affect the physiological function of the cell (Theruvath et al., 2018; Xia et al., 2018). If a magnetic field is applied, each magnetic particle will become a magnetic source, which will enable the magnetic scaffold material to play the role of bone tissue repair therapy. Once the magnetic particles are exposed to an external magnetic field, they will be rapidly magnetized (Yang et al., 2019). The magnetic particles and the magnetic field work together to enhance the effectiveness of their bone tissue repair treatment (Yang et al., 2019; Zhao et al., 2019). Varieties of MNPs loaded with/without drugs have been applied in the medical industry, playing a very important role. Especially for bone tissue repair, MNPs have been proven efficient [Singh et al., 2012a; Maleki, 2014 (Eivazzadeh-Keihan et al., 2019 #160(Eivazzadeh-Keihan et al., 2019 #160)]. This article reviews the common synthesis methods, the mechanism and application of magnetic nanomaterials in the field of bone tissue repair.

## Preparation of Magnetic Nanomaterials

Preparation methods have been well-developed with immeasurable application value. Various elemental compositions have been used in magnetic nanomaterials, including Fe_3_O_4_, Fe, Co, Ni, MgFe_2_O_4_, and Co Fe_2_O_4_ (Hamidian and Tavakoli, 2016; Chen et al., 2018; Wu et al., 2019; Xue et al., 2019). The most classic and common composition of magnetic nanomaterials is Fe_3_O_4_ (Hamidian and Tavakoli, 2016; Yu et al., 2016; Huang et al., 2018). Two main types of magnetic Fe_3_O_4_ preparation methods are the dry method and the wet method (Huang et al., 2018). Among them, the wet method is more commonly applied, mainly including the following techniques, the hydrothermal method, solvothermal method, chemical co-precipitation method, ball milling method, sol-gel method, and the atomic layer deposition method. In the synthesis of MNPs, with different preparation conditions, different preparation methods and different catalysts, turnover number (TON), and turnover frequency (TOF) could differ widely (Singh et al., 2012b; Maleki, 2014, 2018). In the preparation of MNPs for bone repair, with the help of different catalysts, the Suzuki reaction and Heck reaction of halogenated benzene can be carried out efficiently. The overall TON and TOF can reach more than 30,000 mol and 50,000 h^−1^, respectively (Maleki, 2014).

### Organic-Based Method

MNPs can be prepared in sol-gel processes in aqueous solutions, such as co-precipitation methods, microwave synthesis, and hydrothermal reactions (Maleki, 2012, 2013); MNPs can also be prepared in organic solution reactions. Organic phase synthesis has been used to prepare MNPs based on Fe, Co, and Ni alloys, oxides, core/shell, and dumbbell structures (Maleki, 2013, 2014, 2018). The size, shape, and composition of MNPs are affected by one or more reaction parameters, such as reactant concentration, solvent polarity, and reaction temperature/time. Here, we focus on the synthesis of monodisperse MNPs (Table 1).

**Table 1:** Synthesis of monodisperse MNPs.

**Ingredients**	**Reagents**	**Surfactants**	**Solvents**	**Topography & size**	**Magnetic properties**	**Reference**
Fe	Fe(CO)_5_	Oleamine	1-octadecene	Spherical, <10 nm	Ms = 102. 6 emu·g ^−1^	Peng et al., 2006
	Fe(CO)_5_	Oleic acid, oleyl amine	Dioctyl ether	Spherical, 5~ 20 nm	Ms = 173 emu·g ^−1^	Wang G. et al., 2017
	Fe(CO)_5_	Oleamine, cetyl ammonium chloride	1-octadecene	Cube, 15 nm	Ms = 180 emu·g ^−1^	Carlotto et al., 2019
	Fe[N(SiMe_3_)_2_]_2_	2 Oleic acid, cetylamine	Mesitylene	Cube, ~6.2 nm	Mr = 83 emu·g^−1^ Ms = 212 emu·g^−1^	Bodenstein and Eichhofer, 2019
	Fe[N(SiMe_3_)_2_]_2_	Palmitic acid, cetylamine,	Mesitylene	Square, 13–20 nm	–	Carlotto et al., 2019
Co	CO_2_(CO) _8_	Oleic acid, di-n-octylamine	Diphenyl ether	Core-shell structure, spherical, 8–14 nm	Ms = 125 emu·g^−1^	Jiang et al., 2016a
	COCl_2_ (super hydride)	Oleic acid, trialkylphosphine	Dioctyl ether	Spherical nanocrystals, 2~11 nm	Hc = 500 Oe (5K)	Hollingsworth et al., 2018
Ni	NiAc_2_	Oleic acid, tributylphosphine	Diphenyl ether	Spherical, <6 nm	Hc = 3,000 5,000 Oe	Tahir et al., 2016
	Ni(acac)_2_	Oleamine, tri-n-octylphosphine,	Benzyl ether,	Hexagonal superlattice, 4~12 nm	–	Zhang et al., 2020
	Ni(COD)_2_	Cetylamine, tri-n-octylphosphine oxide,	Tetrahydrofuran,	Nanorods, 4 × 15 nm	Ms = 60 emu·g^−1^, Hc = 275 Oe (2K)	Schwab et al., 2018
	Ni(acac)_2_	Tri-n-octylphosphine, cetylamine,	Mesitylene	Nanocube, 12 nm	Hc = 105 Oe (10K), Mr = 3.2 emu·g^−1^	Lan et al., 2017

### Hydrothermal/Solvothermal Method

The hydrothermal method is a widely used synthetic method of magnetic nanocomposites (Gan and Xu, 2019; Pandi et al., 2019; Saygili, 2019). The reaction is generally carried out in a reactor, which includes many advantages, such as simple operation and high reacting efficiency. The metastable phase and nano-morphology obtained by hydrothermal/solvothermal methods are difficult to obtain in other ways (Pandi et al., 2019). Generally, Fe_3_O_4_ is synthesized by the hydrothermal method using FeCl_2_, FeCl_3_, and NaOH at high temperatures in a high-pressure reactor. The reaction principle of the solvothermal method and hydrothermal method is similar, with differences in application of the ethylene glycol medium (Liu et al., 2016). The method is suitable for a wider temperature range as the solvent used has a higher boiling point than water (Pandi et al., 2019). With reducing solvents, products could be protected from oxidation during high-temperature preparation. Researchers have successfully prepared magnetic metal oxides, elemental metals, and alloys using hydrothermal/solvothermal methods (Liu et al., 2016; Wang X. et al., 2018; Saygili, 2019).

### Co-precipitation Method

The most commonly used preparation method of Fe_3_O_4_ is the chemical co-precipitation method (Wan and Li, 2015; Nosrati et al., 2018; Darwish et al., 2019). An alkaline substance is added to the soluble iron salt and ferrous salt solution, creating a precipitate or hydrated precursor (Majidi et al., 2016). Followed by washing, drying, and burning procedures, magnetic nanoparticles would be synthesized. This method is easy to operate and can produce a large number of nanoparticles. The limitation of this method is the poor controllability of its particle size and distribution, because the kinetic factor is the only controllable factor in the grain growth process (Nosrati et al., 2018).

### Ball Milling Method

Mechanical grinding is a method of crushing coarse particles (Fe_3_O_4_ with a general particle size of 10 mm) through strong plastic deformation to the nanometer level (Zhang et al., 2015; Yang et al., 2020). In the planetary ball mill, the coarse-grained material is refined mainly by the collision between the steel balls or between the steel balls and the inner wall of the grinding tank. Using the dry grinding process, nanoparticles can be prepared, but due to repeat crushing-cold welding, the final result is micron-sized particle aggregates with nanograin structures (Narayanaswamy et al., 2019). To obtain highly dispersed nanoparticles, a wet grinding process is required, which has a better grinding and crushing effect. Compared with metal, ceramic powder is more conducive to ball milling due to its brittleness. The physical ball milling method has a good reproducibility of particle size. With expensive equipment, long production cycle, and low efficiency, it is difficult to achieve industrial production (Amiri et al., 2019; Narayanaswamy et al., 2019).

### Sol-Gel Method

The sol-gel method for preparing nano metal oxide is an ideal wet method. This method is based on the hydroxylation and condensation of molecular precursors in solution to form a “sol” of nanoparticles (Kayili and Salih, 2016; Lin et al., 2018a; Wang J. et al., 2018; Bao et al., 2020). Gao, prepared Fe_3_O_4_ nanoparticles under supercritical conditions by the sol-gel method (Gao et al., 2019). The results show that the particles have a monodisperse distribution with an average size of nearly 8 nm. Due to the small particle size and good dispersion, the authors suggested that the particles could be used in biomedical magnetotherapy. Lin et al. reported that monodisperse spherical Co-Cr-Ferrite nanomaterials can be easily prepared by co-condensation of Co(NO_3_)_2_·6H_2_O, chromium nitrate, Cr(NO_3_)_3_·9H_2_O, and iron nitrate using the sol-gel method. Co-Cr-Ferrite nanomaterials were prepared with excellent magnetic properties with stable monodisperse distribution (Lin et al., 2018a).

### Atomic Layer Deposition Method

Recently, atomic layer deposition (ALD) has become an important thin film deposition technology, which is widely used in thin film deposition on the surface of various semiconductors, metal oxides, and polymers (Sanli et al., 2018; Li et al., 2019b; Ponti et al., 2020). This method has many advantages, including precise control of product thickness and excellent uniformity. It is a good method for synthesizing multi-component structure controllable composite materials. Alessandro Ponti synthesized SiO_2_-coated α-Fe_2_O_3_ nanofibers (NFs) with the ALD method (Ponti et al., 2020). It was used for electromagnetic wave absorption. The results showed that the obtained magnetic carbon nanowires have a uniform morphology, which has lower magnetic reflection loss and a higher frequency absorption range than simple carbon nanowires. This means better electromagnetic wave absorption capacity (Ponti et al., 2020).

### Effect of Magnetic Field on Bone Cells

**Figure 1:**
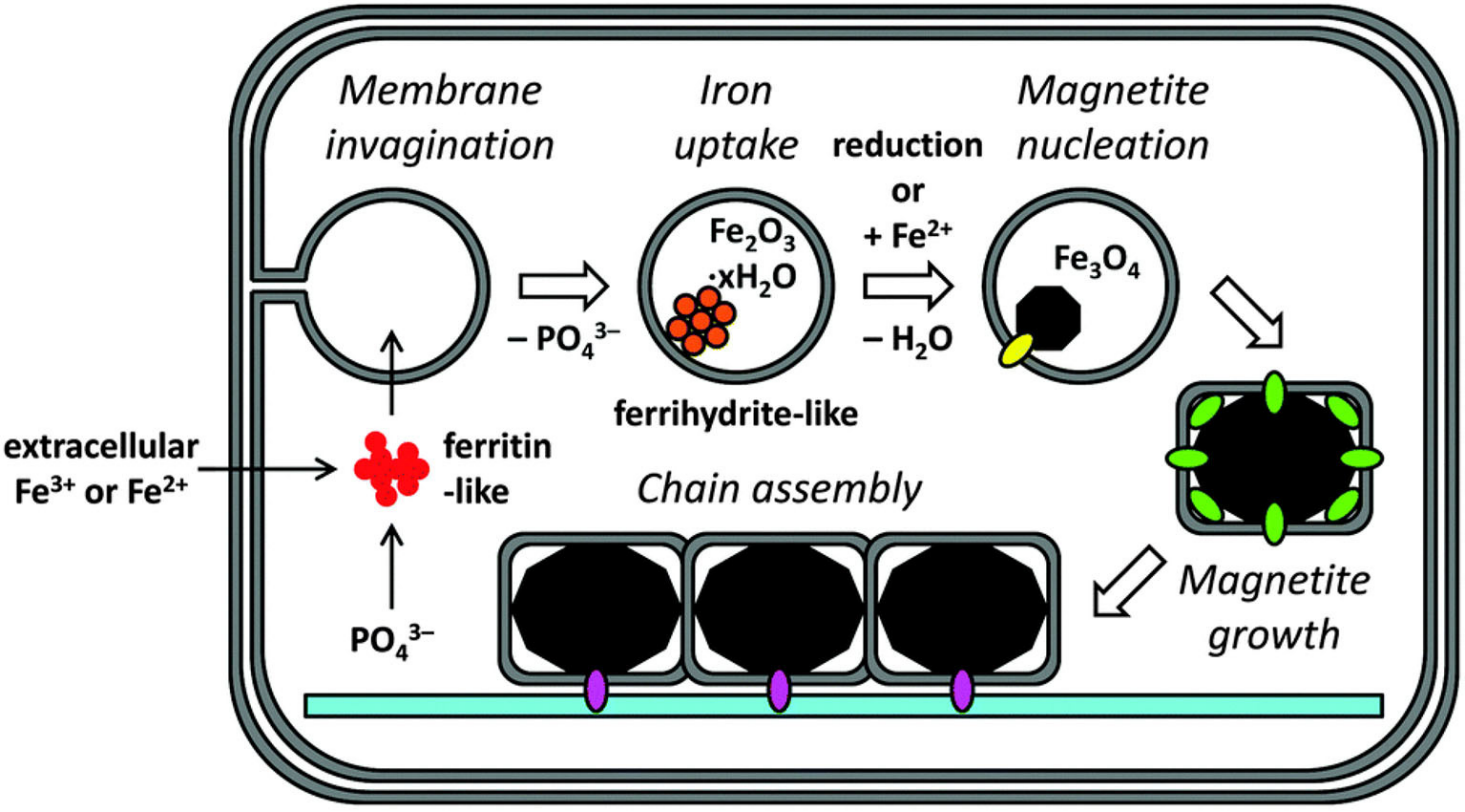
Biomineralizatimodel of magnetite in cellular magnetosomes. Reproduced with permission from Mirabello et al. (2016).

Different magnetic field strengths have different effects on cells, while medium-strength magnetic fields are the most widely used (Mirabello et al., 2016). Recent studies have found that a medium-strength magnetic field can promote the biomineralization process of mouse osteoblasts. The medium-strength magnetic field can promote attachment, proliferation, migration, and the differentiation of cells (Mirabello et al., 2016; Xia et al., 2018). It was confirmed to be related to changes of the calcium ions transport channel on the cell membrane (Rotherham et al., 2018). The magnetic field not only affects the biomineralization behavior in the early stage, but also affects the expression of osteocalcin genes and proteins in the later stage. The production of late stage proteins is proportional to the early stage of biomineralization. A magnetic field affects the structure and crystallinity of biomineralization products and changes the spatial structure of proteins in the cytoskeleton (Figure 1).

**Figure 2:**
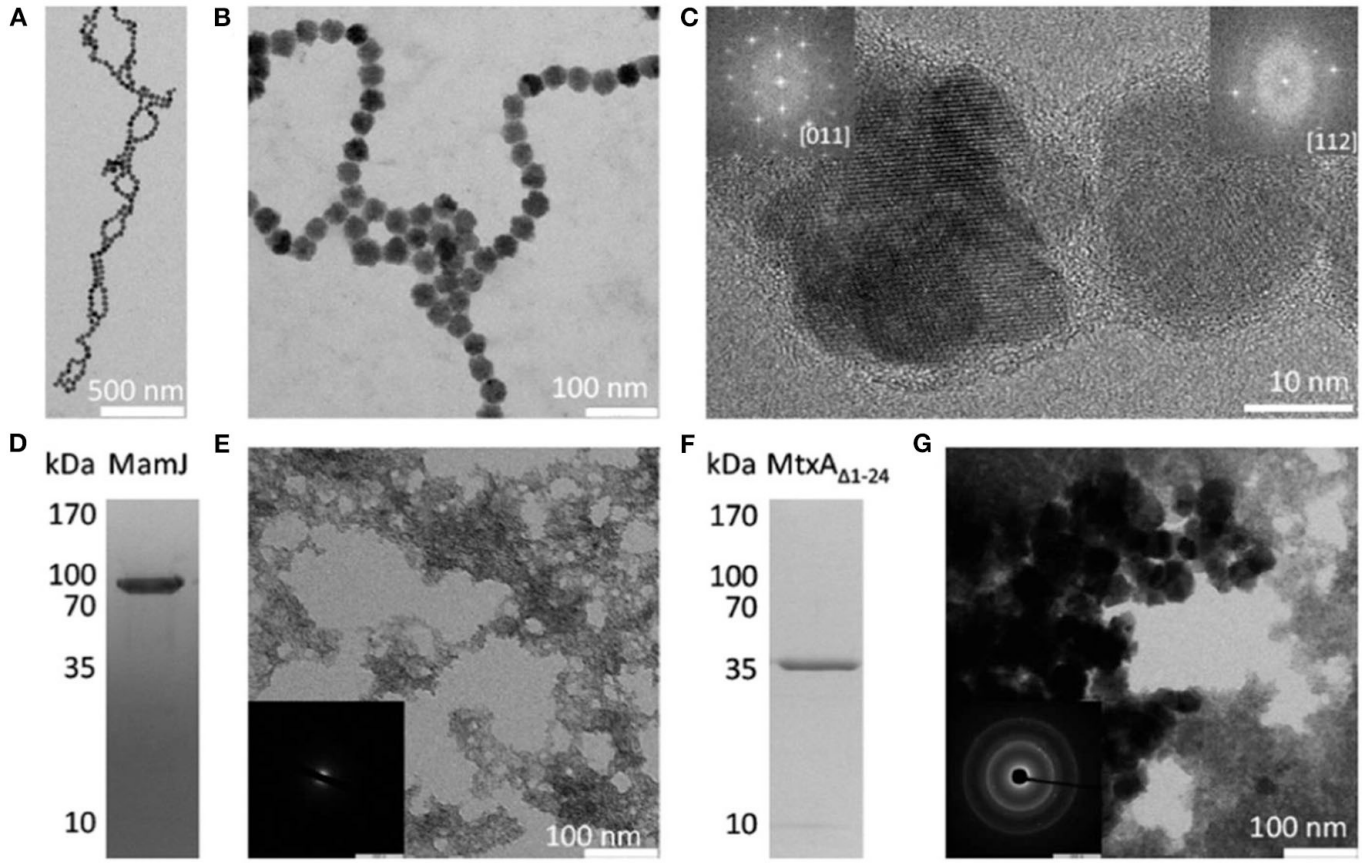
Precipitation products present in polypeptides and proteins showing the reflection with magnetite. **(A–C)** Magnetite particles formed in the presence of poly 1-arginine. The insert in **(C)** the FFT of the particles, showing a reflection consistent with magnetite. **(D)** SDS-PAGE of MamJ. **(E)** Precipitation product in the presence of MamJ. **(E)** an electron diffraction pattern with no signs of crystallization. **(F)** MtxA_Δ1−24_ SDS-PAGE. **(G)** The precipitation product in front of MtxA_Δ1−24_. **(G)** an electron diffraction pattern showing the reflection consistent with magnetite. Reproduced, with permission, from Mirabello et al. (2016).

The magnetic field can be divided into static magnetic field and dynamic magnetic field according to whether the direction of the magnetic field changes (Hu et al., 2015; Abreu et al., 2016). The static magnetic field mainly uses permanent magnets and DC power supplies, while the dynamic magnetic field uses an alternating current (Jiang et al., 2016b). The static magnetic field is divided into four levels according to the strength, the weak magnetic field for <1 mT, the medium magnetic field for 1 mT-1T, the strong magnetic field for 1-5T, and the super magnetic field for > 5T (Mazzone et al., 2017; Wang Z. et al., 2017). Zhang et.al. applied 4 mT electric fields to bone cells. Testing the changes of 16 genes under different magnetic fields by RT-PCR, they found that different magnetic field strengths have different effects on different genes (Zhang et al., 2018a). When the magnetic field is strong, it can change the movement state of the cell (Filippi et al., 2019). Filippi conducted an experiment on osteoblast cells with a strong magnetic field (Filippi et al., 2019). It was found that the cells under the external magnetic field are arranged in the direction of the magnetic field, showing orientation. It was not affected by the orientation of the material surface. The influence of the magnetic field on the cell depends on two aspects (Mirabello et al., 2016). One is the type of cell, the other is the strength and duration of the external magnetic field. The action process of the magnetic field on the cell is shown in Figure 2. Firstly, the cell is attached to the surface of the material and placed on the magnet (Mazzone et al., 2017; Zhang et al., 2018a,b). The direction of the magnetic induction line is perpendicular to the cell. Animal experiments have shown that magnetic fields can promote the attachment, proliferation, differentiation, and expression of growth factors such as bone morphology proteins, accelerate bone fusion, and promote the formation of new bone (Wang Z. et al., 2017; Guerri et al., 2018; Filippi et al., 2019). The magnetic field can also promote the fusion of bone and implant and increase bone density and calcium content to accelerate the healing of bone damage.

### Effect of Magnetic Particles on Bone Repair

**Figure 3:**
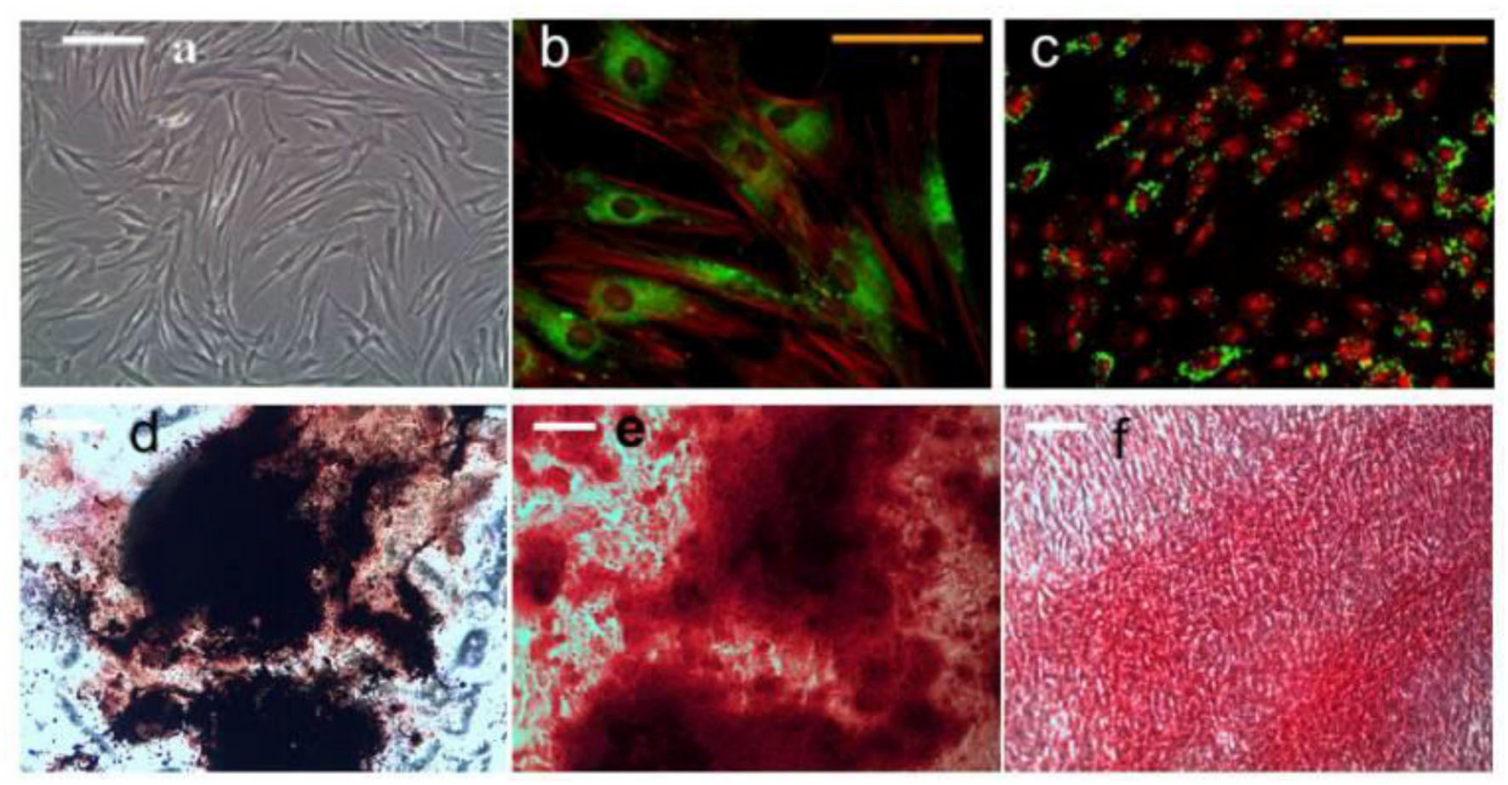
HMSCs treated with magneto mechanical stimulation combined with biochemical factors formed mineralized nodules. Alizarin red and von Kossa co-staining showed positive calcium and phosphorus in the nodules. **(a)** Undifferentiated hMSCs morphology; **(b)** Magnetic nanoparticles (MNP) For PDGFRα; **(c)** integrin ανβ3 hMSCs, **(b,d)** histological analysis of hMSCs after 21 days of osteogenic induction; **(e)** alizarin red s staining; **(f)** Sirius-red staining. Reproduced with permission from Hu et al. (2013).

The magnetic nanoparticles have the ability to bind to the cell surface, which makes it possible to control and regulate the function of cells under the condition of an external magnetic field (Hurle et al., 2018; Fernandes et al., 2019; Parfenov et al., 2020). Qian et al. studied the effect of magnetic nanoparticles on bone marrow mesenchymal stem cells (Qian et al., 2018). The results show that magnetic nanoparticles are accumulated in mesenchymal stem cells at an average concentration of 20 pg per cell, which has no adverse effect on the proliferation and differentiation of bone marrow mesenchymal stem cells (Qian et al., 2018). Under the action of an external magnetic field, magnetic nanoparticles can significantly promote the proliferation of bone marrow mesenchymal stem cells. Dabrowska studied the effects of magnetic nanoparticles and an applied magnetic field on human mesenchymal stem cells (Ahn et al., 2018; Dabrowska et al., 2018). After 21 days of cultivation *in vitro* with induction medium, human mesenchymal stem cells have the ability to differentiate into osteoblasts, adipocytes, and chondrocytes under the action of magnetic nanoparticles with the magnetic field (Hu et al., 2013; Yang et al., 2018) (Figure 3). Cells cultured *in vitro* were collected and implanted in the skull bone defect of nude mice (Mortimer and Wright, 2017; Qian et al., 2018). Histological observation confirmed that there was obvious new bone formation 14 days after the cells were implanted, while no new bone formation was found in the control (no implanted cells). It showed that the combined action of magnetic nanoparticles and the magnetic field can promote bone repair (Tang et al., 2017; Yang et al., 2018). Magnetic nanoparticles have been proven to promote bone tissue regeneration mainly through the magnetic force generated by itself and the external magnetic field (Mortimer and Wright, 2017; Lin et al., 2018b; Fernandes et al., 2019). Fernandes et al. studied the effect of an external magnetic field on bone repair. They implanted calcium phosphate-magnetic nanoparticle composites under the back of a rat (Hurle et al., 2018; Qian et al., 2018; Fernandes et al., 2019). Under the condition of an external magnetic field, this composite material can significantly promote the proliferation and differentiation of cells and 30 days after the formation of new bone tissue. This shows that the magnetic field can promote bone repair (Tang et al., 2017; Ahn et al., 2018; Yang et al., 2018).

In order to ensure the full effect, it is necessary to maintain the distribution of nanoparticles among MNPs. The proportion of mismatched atoms on the surface of the material increases as the particle size decreases, while this degree of mismatch is more pronounced at the nanometer scale (Maleki, 2013, 2014). Between nanoparticles, the effects of the chemical bonding force, van der Waals dispersion force, and Coulomb force are very strong. Particles are very easy to reunite. From the perspective of surface energy, most MNPs are hydrophobic with large surface energy (Singh et al., 2012a; Singh and Kim, 2013). In order to minimize the overall surface energy, these nanoparticles are prone to agglomeration, forming large agglomerates, which ultimately leads to an increase in particle size. According to the agglomeration mechanism of MNPs, the dispersion problem of nanometers is mainly to eliminate the problem of hard agglomeration (particles agglomerated in a face-to-face connection) between nanoparticles (Singh et al., 2012a). At present, widely used anti-agglomeration methods include organic solvent washing, freeze drying, anti-agglomeration under ultrasonic action, and surfactant anti-agglomeration. Among them, surfactant which is also known as surface modifier, is the most effective in preventing agglomeration. When MNPs reunite, degrade, or change in structure, their functions and bone repair effects under magnetic fields will change accordingly (Singh and Kim, 2013; Eivazzadeh-Keihan et al., 2019). Compared with the MNPs in good condition, the particles with structural changes mainly show that the responsiveness of the magnetic field stimulus decreases, the activity of bone cells decreases, and the bone repair function decreases after the action (Maleki, 2012; Singh et al., 2012b, 2014).

### Magnetic Nanomaterial Bone Repair Application

Magnetic nanomaterials not only have the unique properties of nanoparticle materials, but also have magnetic responsiveness and superparamagnetism (Xia et al., 2018; Fernandes et al., 2019). They can gather and position under a constant magnetic field and absorb electromagnetic waves to generate heat under an alternating magnetic field (Li et al., 2016). Among them, magnetic iron oxide nanoparticles are widely used in magnetic stimulation to promote bone formation, drug loading, bone formation with stem cells, and bone formation with scaffolds (Yun et al., 2016a; Rotherham et al., 2018; Xia et al., 2018, 2019a; Zhao et al., 2019). Magnetic nanomaterials have shown good bone-promoting effects in many studies and have good application prospects (Lu et al., 2018; Fernandes et al., 2019).

### Magnetic Nanoparticles Combined With Magnetic Field Stimulation

**Figure 4:**
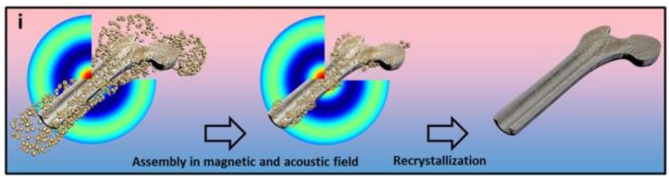
The schematic representation of the fabrication of a three-dimensional scaffold using a magnetoacoustics' field, including the assembly and recrystallization of the scaffold. Fabrication of calcium phosphate 3D scaffolds for bone repair using magnetic stimulation. Reproduced with permission from (Parfenov et al., 2020).

Bigham et al. grafted a magnetic Mg_2_SiO_4_-CoFe_2_O_4_ composite scaffold on the surface of warp-based apatite particles. Compared with the traditional HA particles, FeHA improves the survival rate of osteoblasts under the effect of an external magnetic field and affects the cell morphology (Bigham et al., 2019). Parfenov et al. used magnetic particles and apatite as bone tissue engineering scaffolds. Under the action of an external magnetic field, osteoblasts will show a positive effect on the proliferation and differentiation. Both the magnetic particles and the applied magnetic field have a synergistic effect on the cells (Parfenov et al., 2020) (Figure 4).

One disadvantage of magnetic particles is their easy agglomeration, which limits their application in the field of biomedical medicine (Luo et al., 2018; Rotherham et al., 2018). By using chemical co-precipitation method, MNPs were evenly dispersed into PVA nanofibers, and then made into a film by *in-situ* generation and an electrospinning method to obtain PVA nanofiber membranes containing MNPs with good dispersion (Manjua et al., 2019). Parfenov et al. grew osteoblasts on the surface of polystyrene, and applied a static magnetic field of 150 mT, which can promote the proliferation and differentiation of osteoblasts (Parfenov et al., 2020). Fernandes discovered that magnetic fields can promote gene expression in mouse neural stem cells (Fernandes et al., 2019). Rotherham found in their research that the magnetic field not only affects the synthesis of DNA and RNA of cells, but also affects the movement state and morphology of cells when the magnetic field strength is > 10T (Rotherham et al., 2018). Díaz prepared PLLA nanofiber membrane by electrospinning. When conducting *in vitro* cell culture experiments, it was found that the orientation of the nanofiber membrane itself had a contact guiding effect on osteoblasts (Díaz et al., 2019).

### Magnetic Nanoparticle Drug Loading

**Figure 5:**
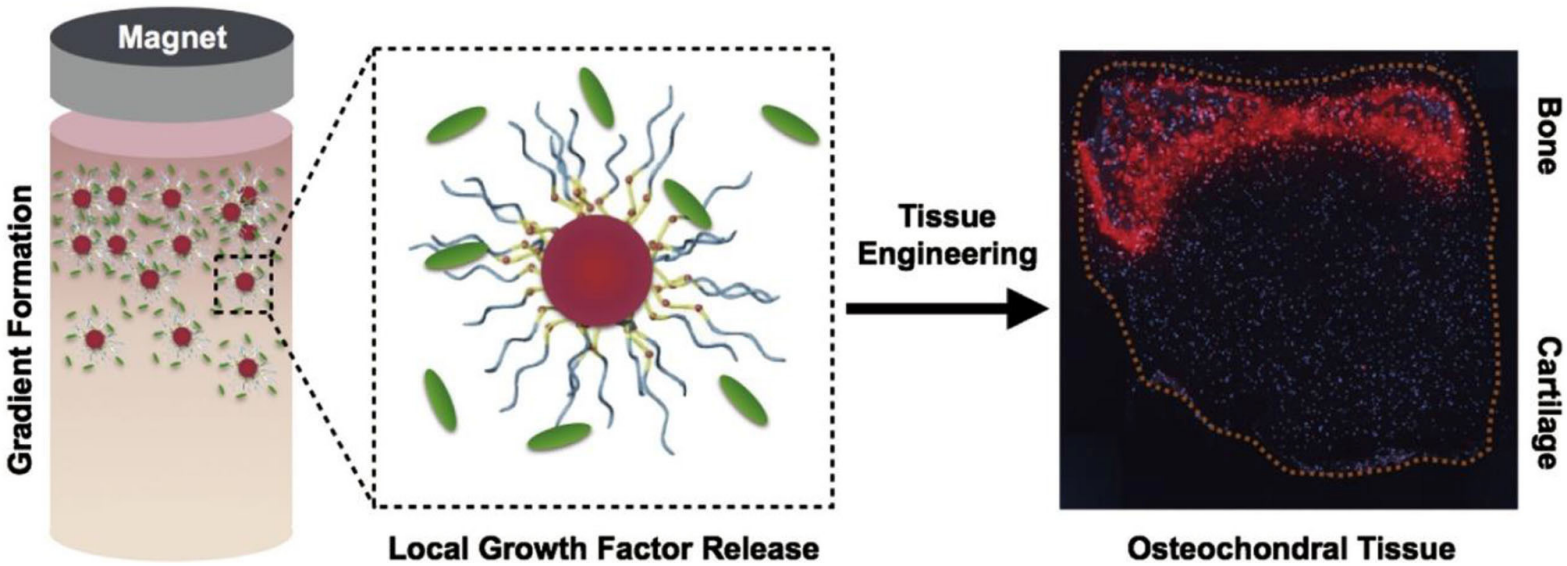
The use of magnetically aligned glycosylated cations to produce osteochondral tissue. The coupling of anions with heparin produces a glycosylated canopy that can effectively isolate and release growth factors, the key mineralized protein osteopontin (Iwasa & Reddi), which is specifically present at the bone end of tissues. Reproduced with permission from Li et al. (2018).

“Drug targeted therapy” is a way to make drugs selectively enriched in the pathological site for treatment (Kumari et al., 2016; Shen et al., 2016; Jung et al., 2018). Compared with the traditional method of administration, it can reduce the dose of drugs, reduce the possible side effects of the drugs on healthy tissues, and greatly reduce the cost of drug treatment (Kumari et al., 2016; Qi et al., 2016; Sun et al., 2019). The structure of the magnetic microsphere loaded with release growth factors is shown in Figure 5, in which the magnet is served by nano-magnetic particles (Li et al., 2018).

Antibiotics, growth factors, and microRNA and siRNA related to osteogenesis can improve the repair effect of bone defects (Jia et al., 2018; Li et al., 2018; Ferjaoui et al., 2019). However, these drugs cannot target specific sites or produce a direct repair effect. In recent years, people have been working on finding the best drug delivery system to reduce the adverse effects and toxicity of drugs, reduce the loss of drug efficacy, and improve the therapeutic effect (Jung et al., 2018; Lugert et al., 2019; Sun et al., 2019; Vangijzegem et al., 2019). Studies found that iron oxide nanoparticles can be used as a means of delivery, which can effectively carry drug molecules under the action of an external magnetic field and target specific parts of the body (Denyer et al., 2018). Suspension of drug molecules on magnetic carriers or dispersion on magnetic nanoparticles is a simple and direct magnetic targeted drug delivery route (Denyer et al., 2018). Recent studies have used solvent evaporation and lyophilization to prepare degradable polylactic acid-glycolic acid copolymer capsaicin-coated magnetic nanoparticles (Xia et al., 2018). This magnetic nanoparticle provides the continuous release of capsaicin. Under the action of an external magnetic field, the magnetic nanoparticles can reach specific locations and improve the therapeutic effect on specific locations (Xia et al., 2018). In addition to magnetic targeting, magnetic heating is also used to control the release of therapeutic drugs from thermally responsive drug carriers. Xia et al. coated iron oxide nanoparticles with bisphosphonate and dextran to obtain bisphosphonate/dextran/Fe_3_O_4_ nanoparticles, which were thermally decomposed using a radio frequency system. Devouring has a strong destructive effect (Xia et al., 2019a). In the other study, magnetic multi-walled carbon nanotubes, hydroxyapatite, and clodronate were synthesized into nanocomposites. It was found that clodronate can be continuously released from the system and inhibit the formation of osteoclasts (Swietek et al., 2019). The magnetic liposomes are obtained by embedding iron oxide nanoparticles into the liposome membrane. When an alternating magnetic field is applied externally, the generated heat will destroy the cell membrane, releasing the drug encapsulated in the liposome for therapeutic purposes. In addition to promoting osteogenic differentiation, iron oxide nanoparticles also have good bone conduction ability (Xia et al., 2018; Swietek et al., 2019). Currently in the field of bone regenerative medicine, iron oxide nanoparticle complexes are often used as carriers for the controlled release of drugs. Loading gentamicin in multifunctional magnetic mesoporous bioactive glass has been studied. The Fe_3_O_4_ nanoparticles in multifunctional magnetic mesoporous bioactive glass improved the continuous release of gentamicin, which is helpful to reduce the adhesion of bacteria and prevent biofilm formation (Wang et al., 2015). In addition to treating infection, the Fe_3_O_4_ nanoparticles in the composite can also promote the adhesion, proliferation, and osteogenic differentiation of bone marrow mesenchymal stem cells (Xia et al., 2018; Swietek et al., 2019). Nanomaterials have been used as carriers to deliver therapeutic drugs in cells, including proteins, growth factors, small molecule chemicals, and DNA/RNA (Cruz-Acuña et al., 2018; Kuai et al., 2018; Xia et al., 2018, 2019a). This magnetic drug delivery system will provide a useful support platform for bone defect repair.

### Magnetic Nanoparticles With Stem Cells

**Figure 6:**
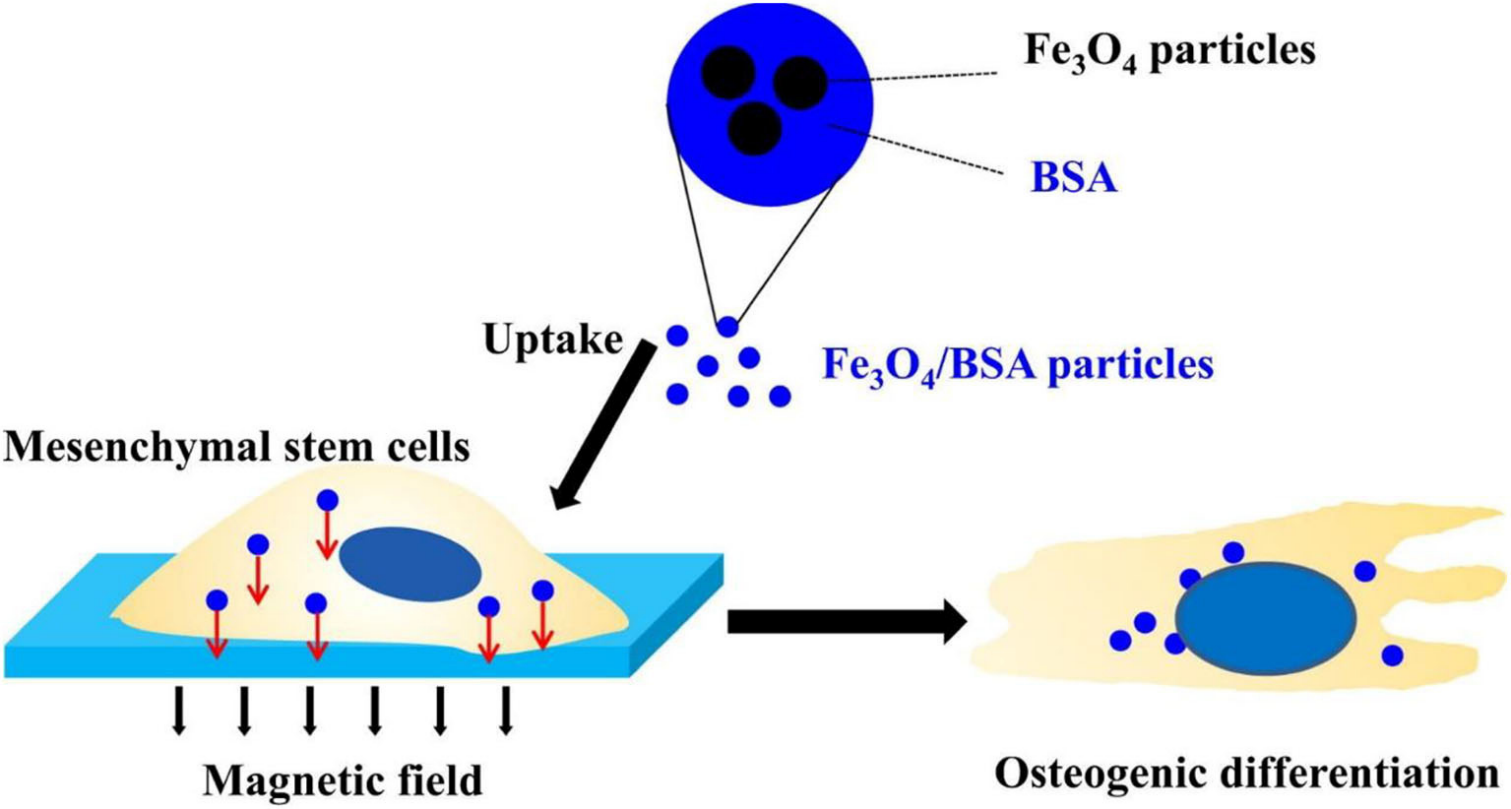
Fe_3_O_4_/BSA particles significantly promote the osteogenesis of mesenchymal stem cells under the action of an external magnetic field, while magnetic particles and an external magnetic field alone do not affect the differentiation of stem cells. Reproduced with permission from Liu et al. (2016).

In recent years, stem cell therapy has been proffered as a strategy for repairing bone defects, especially in repairing large area bone defects (Xia et al., 2018; Li et al., 2019a). At present, although stem cell transplantation has been successful in treating animal bone defect models, these traditional stem cell transplantations have not achieved similar results in clinical treatment (Xia et al., 2018). How to maintain long-term and effective stem cell characteristics is the key to successful treatment. Therefore, it is necessary for bone expansion to maintain a stable cell phenotype and reduce cell necrosis at the defect site after transplantation *in vivo* (Liu et al., 2018; Nejadnik et al., 2019). With the development of materials science and chemical biology, researchers have tried to use iron oxide nanoparticles as a tool for the research and control of stem cells for many years (Li et al., 2019a). Iron oxide nanoparticles can be combined with an external magnetic field to affect cell adhesion, proliferation, movement and distribution, and stem cell osteogenic differentiation. In addition, iron oxide nanoparticles can be used to label cells for *in vivo* tracking and monitoring (Son et al., 2015; Nejadnik et al., 2019). Liu et al. found the mechanism of Fe_3_O_4_/BSA particles uptake into stem cells, promoting the osteogenesis under the action of an external magnetic field (Liu et al., 2016) (Figure 6).

One of the applications of iron oxide nanoparticles in stem cell therapy is to magnetically target stem cells to a suitable location (Khan et al., 2018; da Silva et al., 2019; Jia et al., 2019). In some studies, an *in vitro* magnetic targeting system was developed to attract rabbit bone marrow mesenchymal stem cells. It was found that this technology significantly promoted cells penetration and bone formation labeled with iron oxide nanoparticles into porous hydroxyapatite ceramics transplanted into rabbit ulnar defects (Li et al., 2019a). Similarly, Khan et al. also reported that a magnetite cationic liposome (MCLS) with Fe_3_O_4_ nanoparticles as the core and columnar neodymium magnets were used to expand mesenchymal stem cells (Khan et al., 2018). After culturing, the density and number of mesenchymal stem cells were significantly higher than those of conventional cultured cells. It revealed that iron oxide nanoparticles can attract stem cells to homing. They can also promote the differentiation of mesenchymal stem cells *in vitro* and *in vivo* (Khan et al., 2018). Son et al. used magnetically labeled mesenchymal stem cells to study a magnetic targeting system for repairing severe chronic bone defects using external magnetic devices (Son et al., 2015). It was found that transplanting with enough stem cells can completely repair severe chronic bone defects. In addition to the strategy of promoting bone regeneration by controlling stem cells, iron oxide nanoparticles themselves, especially under the stimulation of an external magnetic field, can induce the osteogenic differentiation of stem cells (Son et al., 2015). Jia et al. prepared polydextrose sorbitol carboxymethyl ether coated iron oxide nanoparticles and studied their effects on bone marrow mesenchymal stem cells (Jia et al., 2019). The results showed that iron oxide nanoparticles were structurally stable in bone marrow mesenchymal stem cells promoting the osteogenic differentiation of bone marrow mesenchymal stem cells. Gene microarray analysis and bioinformatics analysis showed that iron oxide nanoparticles can activate the classic mitogen-activated proline kinase (MAPK) signaling pathway (Sweeney et al., 2018; Xia et al., 2018). Under a static magnetic field, Fe_3_O_4_ particles can significantly promote the expression of alkaline phosphatase, type I collagen and osteocalcin at the mRNA, and protein levels in mesenchymal stem cells (Liu et al., 2018; Xia et al., 2018). The magnetic effect of the nanoparticles with mesenchymal stem cells have preliminarily demonstrated that iron oxide nanoparticles have great potential in bone regeneration and repair.

### Magnetic Nanoparticles and Tissue Engineering Scaffolds

**Figure 7:**
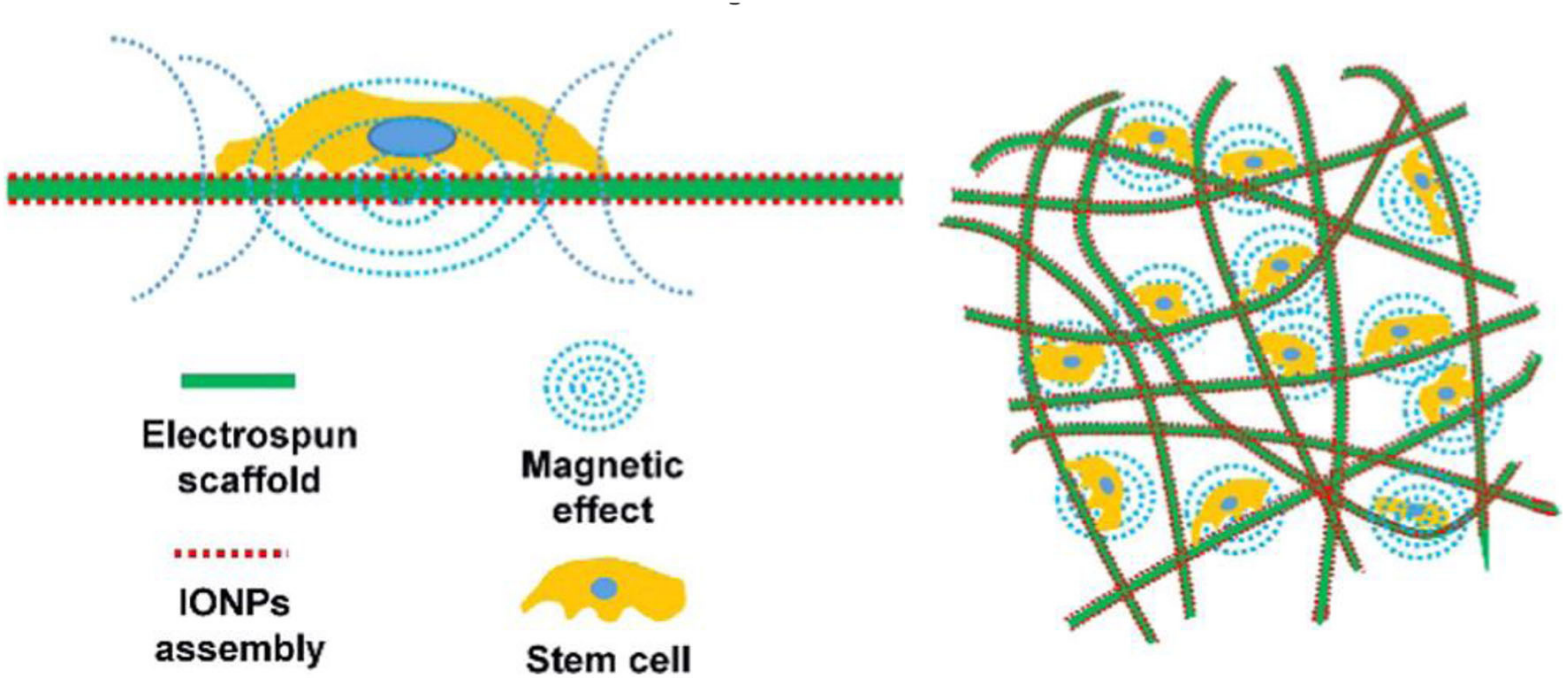
The mechanism of ion assembly electrospinning scaffold on cells is shown. The key lies in the local magnetic field. Magnetization promotes osteogenic differentiation of stem cells. Reproduced with permission from Chen et al. (2018).

Tissue engineering is a method of rebuilding functional tissue at the damaged site, usually including seed cells, growth factors, and three-dimensional biodegradable scaffolds (Cruz-Acuña et al., 2018; Kuai et al., 2018; Luo et al., 2018; Cojocaru et al., 2019; Li et al., 2020). The construction of a tissue engineering complex is of great significance to promote tissue regeneration or healing. The current problem is that cells usually stay on the surface of the material and cannot enter the scaffold. Recent studies have found that cells can be driven to the center of the three-dimensional scaffold with the help of magneto-mechanical drive. Parfenov et al. reported a technique of using magnetic force to seed cells, using a porcine acellular common carotid artery stent (Eivazzadeh-Keihan et al., 2020; Parfenov et al., 2020). The porcine acellular common carotid artery stent was immersed in a suspension of magnetically labeled cells. Attached to the porcine acellular common carotid artery stent, it was found with the absorption of iron oxide nanoparticles, the cell adhesion gradually increased. In addition, some studies have found that iron oxide nanoparticles coated with chitosan can enhance the depth of human osteoblasts into the 3D scaffolds. It can take advantage of the magnetic force to increase the interaction between cells and shorten the cell proliferation cycle (Eivazzadeh-Keihan et al., 2019; Parfenov et al., 2020). The application of this magnetic technology has important value in promoting bone tissue repair and regeneration. It could also provide an important theoretical basis for researchers and clinicians (Mahdavinia et al., 2018; Manjua et al., 2019; Parfenov et al., 2020). In recent years, based on the application of magnetic tissue engineering, multi-layer cell membranes have been developed, which will greatly promote the repair of bone tissue. In addition, some studies have found that magnetic tissue engineering can not only induce the formation of multifunctional stem cell membranes, but also promote the formation of repairing blood vessels [(Kim et al., 2014) (Singh et al., 2014#170 (Singh et al., 2014#170; Yun et al., 2016b)]. The application of magnetic tissue engineering can provide a new method for bone tissue engineering. Chen et al. studied the mechanism of ion assembly electrospinning scaffold on cells (Chen et al., 2018) (Figure 7). They found that magnetization promotes osteogenic differentiation of stem cells due to the local magnetic field.

The magnetic scaffold can attract growth factors and stem cell migration in the body through magnetic drive, promoting bone repair and regeneration (Díaz et al., 2018; Xia et al., 2018). At present, the role of magnetic scaffolds in promoting cell proliferation and new bone tissue growth has been confirmed (Lu et al., 2018). The scaffold has a wide range of components, mainly including biological macromolecules, synthetic polymers, polyethylene glycol, and inorganic materials (Dashnyam et al., 2014; Cruz-Acuña et al., 2018; Kuai et al., 2018; Luo et al., 2018; Cojocaru et al., 2019; Liu Z. Y. et al., 2020).

**Figure 8:**
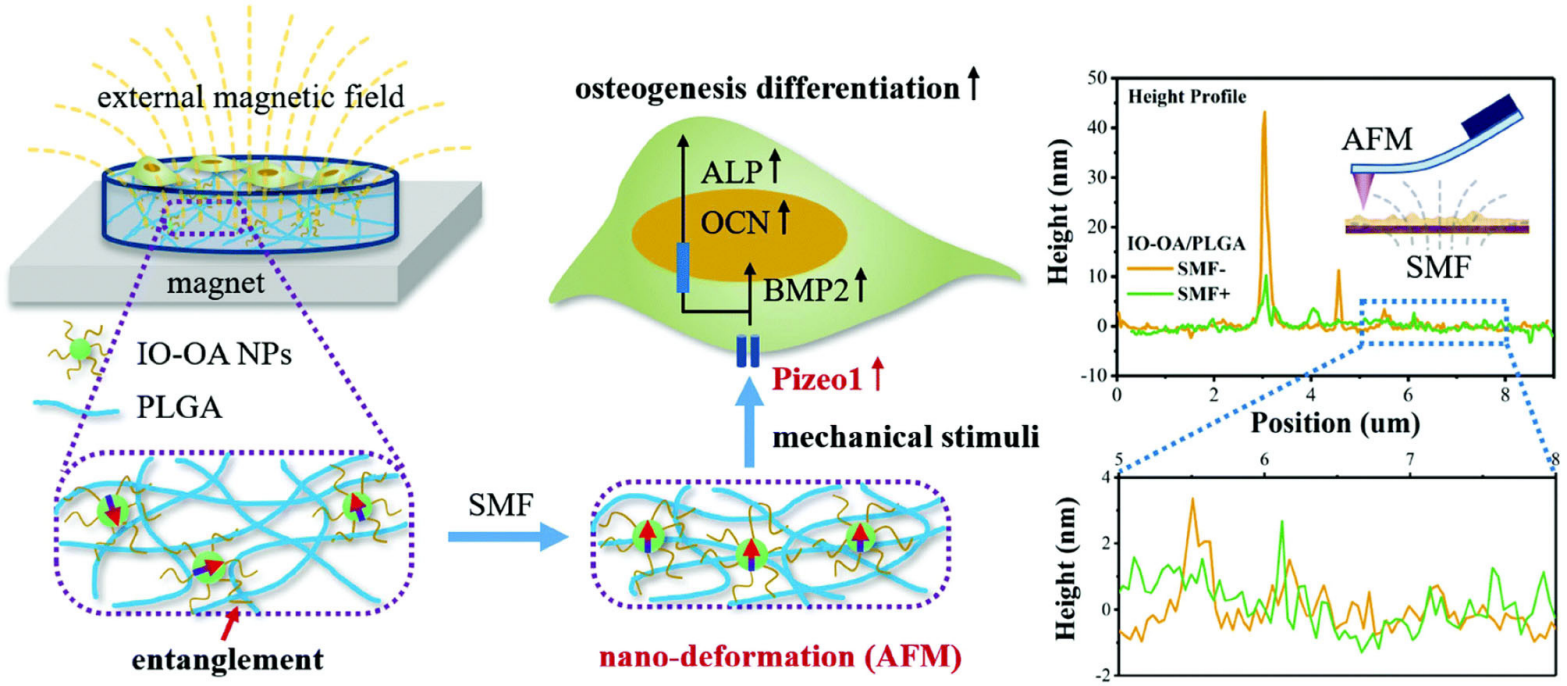
Schematic diagram shows that the magnetic mechanical stimulation caused by the nano-deformation of IO-OA/PLGA nanocomposites under SMF leads to the enhanced osteogenic differentiation of MC3T3-E1 cells. Reproduced with permission from Gao et al. (2019).

It was found that magnetic hydroxyapatite scaffolds loaded with superparamagnetic iron oxide nanoparticles (SPIONs) hydroxyapatite can significantly improve the adhesion and proliferation of bone cells (Yun et al., 2016b; Díaz et al., 2018; Xia et al., 2018). In addition, some studies have found that under the action of a static magnetic field, the magnetic composite scaffold material can obviously promote the proliferation, differentiation, and extracellular matrix secretion of MC3T3-E1 cells (Díaz et al., 2018; Luo et al., 2018). Similarly, Fernandes et al. prepared magnetic nanocomposite scaffolds of iron oxide nanoparticles and polycaprolactone (Fernandes et al., 2019). They found that compared with pure polycaprolactone scaffolds, the magnetic nanocomposite scaffolds had significant advantages in cell adhesion, alkaline phosphatase activity, and expression of genes related to osteogenesis (Fernandes et al., 2019). In addition, the iron oxide nanoparticle nanofiber scaffold can promote bone regeneration of radial segmental defects. Gao et al. applied nano-deformation of IO-OA/PLGA nanocomposites under SMF (Gao et al., 2019). They found that the magnetic mechanical stimulation led to the enhanced osteogenic differentiation of MC3T3-E1 cells (Gao et al., 2019) (Figure 8).

**Figure 9:**
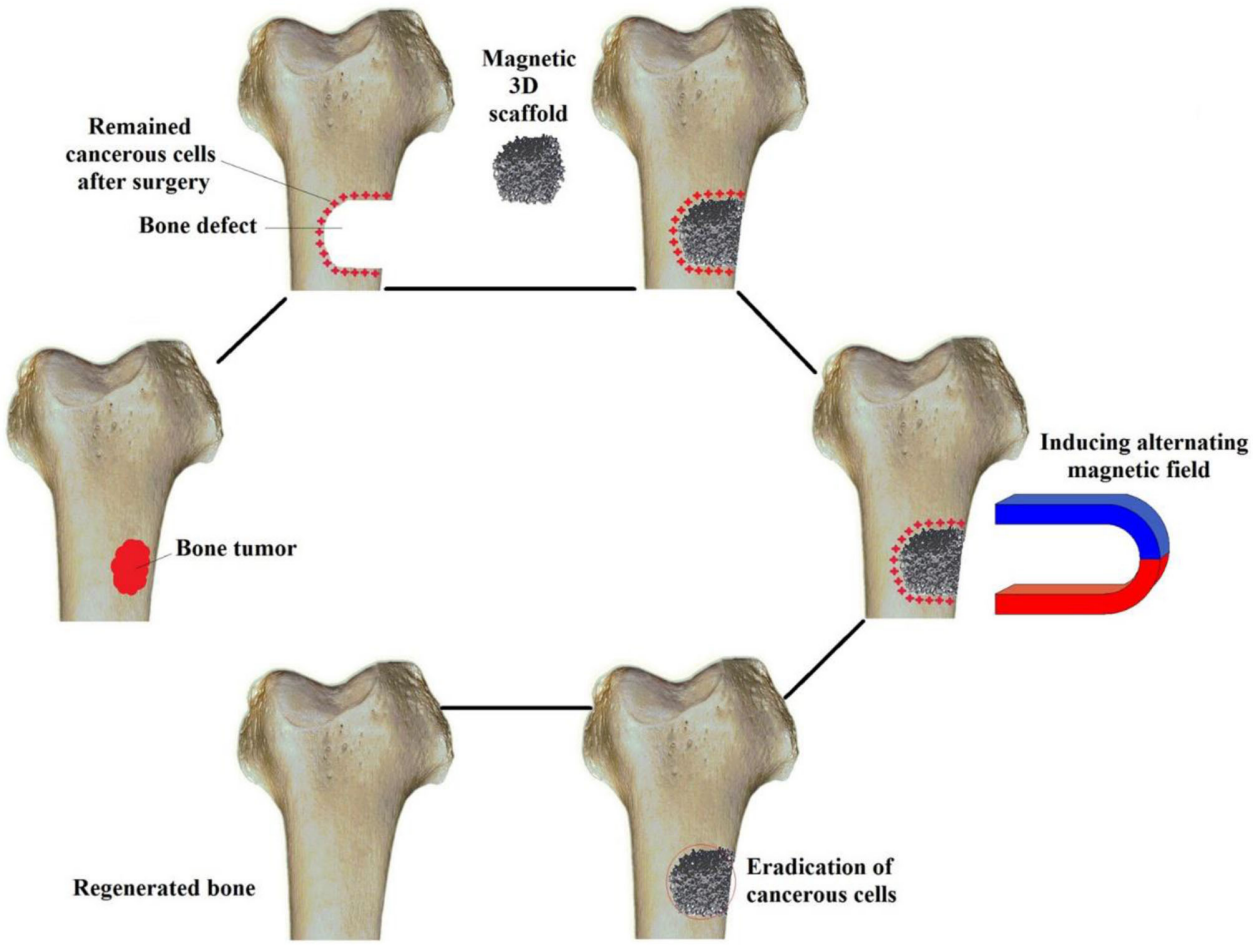
Schematic for applying a Mg_2_SiO_4_-CoFe_2_O_4_ nanocomposite scaffold as a multifunctional magnetic scaffold to eradicate remaining bone cancerous tissues after surgery and regenerate the defect. Reproduced with permission from Bigham et al. (2019).

The potential mechanism of iron oxide nanoparticles to enhance bone formation *in vivo* and *in vivo* is still being explored (Yun et al., 2016a; Luo et al., 2018; Xia et al., 2019b). Xia et al. found *in vivo* experiments that compared with the pure hydroxyapatite scaffold, the concentration of calcium ions and G protein-coupled receptors on the magnetic hydroxyapatite scaffold increased (Xia et al., 2019b). It was found that functional protein enriched on the magnetic scaffolds can effectively enhanced the osteogenic activities of stem cells through WNT/β-catenin signaling, resulting in increased proliferation of MC3T3-E1 cells and accelerated osteogenic differentiation. Cojocaru et al. also studied the combined effect of Calcium phosphates composites with inclusions of magnetic nanoparticles for bone tissue engineering. They found that a static magnetic field and magnetic scaffold can promote osteogenic differentiation of mouse skull osteoblasts and enhance bone related genes expression and alkaline phosphatase activity (Cojocaru et al., 2019). Although more and more experimental data confirmed that iron oxide nanoparticles have obvious effects on the survival and differentiation of bone cells, especially under the external magnetic field. The interaction with bone cells and magnetic mechanical stimulation still needs further study. Bigham applied a Mg_2_SiO_4_-CoFe_2_O_4_ nanocomposite scaffold as a multifunctional magnetic scaffold to eradicate remaining bone cancerous tissues after surgery (Bigham et al., 2019) (Figure 9). It is a promising attempt for bone tissue repair and defect regeneration with magnetic nanomaterials along with a 3D scaffold. It is deemed to potentially help solve clinical bone repair problems in the future.

## Conclusion

This paper reviews advances on the application of magnetic nanomaterials in osteogenesis repair. The application of magnetic nanomaterials in bone regeneration has opened up a new way to repair bone defects. The unique properties of magnetic nanomaterials have accelerated their application in medicine, especially in terms of magnetism, magnetic fields can provide remote control of drug release and biomolecule activation, generating biological reactions including cell differentiation, tissue growth, and bone defect regeneration. The development of a magnetic field suitable for a bone repair application will facilitate the safe, convenient, and effective control of magnetic nanomaterials. Although the application of magnetic nanomaterials in bone regeneration has achieved initial success, the interaction mechanism between magnetic nanomaterials, magnetic fields, and osteoblasts is not yet clear, and the mechanism and principle of action need to be clarified in the future.

## Author Contributions

DaF, QW, and TZ contributed equally to this reviewed paper. XL, DoF, and XW conceived and designed the content of the paper. DaF, QW, and BL collected the researched literatures, arranged the outline of collected documents, and wrote the articles. HW, BL, YW, and ZL made important suggestions and helped revising the paper. All authors reviewed and commented on the entire manuscript.

## Conflict of Interest

The authors declare that the research was conducted in the absence of any commercial or financial relationships that could be construed as a potential conflict of interest.
